# Voltammetric Sensor Based on the Poly(*p*-aminobenzoic Acid) for the Simultaneous Quantification of Aromatic Aldehydes as Markers of Cognac and Brandy Quality

**DOI:** 10.3390/s23042348

**Published:** 2023-02-20

**Authors:** Guzel Ziyatdinova, Tatyana Antonova, Rustam Davletshin

**Affiliations:** 1 Analytical Chemistry Department, Kazan Federal University, Kremleyevskaya 18, Kazan 420008, Russia; 2 Department of High Molecular and Organoelement Compounds, Kazan Federal University, Kremleyevskaya 18, Kazan 420008, Russia

**Keywords:** voltammetric sensors, chemically modified electrodes, electropolymerization, aminobenzoic acid, carbon nanotubes, syringaldehyde, vanillin, cognac and brandy, food quality

## Abstract

Cognac and brandy quality control is an actual topic in food analysis. Aromatic aldehydes, particularly syringaldehyde and vanillin, are one of the markers used for these purposes. Therefore, simple and express methods for their simultaneous determination are required. The voltammetric sensor based on the layer-by-layer combination of multi-walled carbon nanotubes (MWCNTs) and electropolymerized *p*-aminobenzoic acid (*p*-ABA) provides full resolution of the syringaldehyde and vanillin oxidation peaks. Optimized conditions of *p*-ABA electropolymerization (100 µM monomer in Britton–Robinson buffer pH 2.0, twenty cycles in the polarization window of −0.5 to 2.0 V with a potential scan rate of 100 mV·s^−1^) were found. The poly(*p*-ABA)-based electrode was characterized by scanning electron microscopy (SEM), cyclic voltammetry, and electrochemical impedance spectroscopy (EIS). Electrooxidation of syringaldehyde and vanillin is an irreversible two-electron diffusion-controlled process. In the differential pulse mode, the sensor allows quantification of aromatic aldehydes in the ranges of 0.075–7.5 and 7.5–100 µM for syringaldehyde and 0.50–7.5 and 7.5–100 µM for vanillin with the detection limits of 0.018 and 0.19 µM, respectively. The sensor was applied to cognac and brandy samples and compared to chromatography.

## 1. Introduction

Aged distilled beverages (cognac and brandy among them) contain various types of natural phenolic antioxidants such as aromatic aldehydes and phenolic acids [1]. Aromatic aldehydes determine the aroma, smell, and taste of the beverage and their contents and concentration ratio can be used as markers of cognac and brandy quality [2,3,4,5]. Syringaldehyde and vanillin, as the products of oak-wood lignin destruction during ethanolysis [6], are major aromatic aldehydes and markers of the beverage quality [2,3]. The ratio of syringaldehyde and vanillin concentrations in cognac and brandy is varied in the range of 1.4–3.0 depending on the age of the alcohols used [5]. In the case of adulteration, vanillin is usually added to imitate the organoleptic properties [3] that leads to a change in this ratio and can serve as one of the diagnostic criteria of aged distilled beverages quality. The concentration ranges of 0.20–34.20 mg·L^−1^ for syringaldehyde and 0.10–18.40 mg·L^−1^ for vanillin are reported and depend on the origin and denomination of the beverage [7].

Cognac and brandy are characterized by a complex chemical composition, which imposes high requirements on the selectivity of the analytical methods used. In this case, direct simultaneous determination of the structurally related compounds is an important problem, which is traditionally solved using separation and concentration methods, in particular, various types of chromatography and electrophoresis.

Simultaneous quantification of syringaldehyde and vanillin has been successfully achieved using high-performance liquid chromatography (HPLC) [8,9,10,11] and ultra-HPLC [12,13,14,15] with UV- [8,12], diode-array [9,10,11], and tandem mass-spectrometric [13,14,15] detection. Capillary electrophoresis with UV-detection [16] and high-performance capillary electrophoresis with diode-array detection [2,17] have been developed for the simultaneous determination of syringaldehyde and vanillin in cognac, brandy, and aged wine distillates. Original approaches to the electrophoretic determination of aromatic aldehydes are based on the application of online preconcentration using normal sample stacking and sample stacking with matrix removal [18] or field-amplified sample stacking [19]. However, the development of more simple, cost-effective, and express methods is encouraged.

Electrochemical sensors are a good alternative to chromatography and electrophoresis because of their simplicity, rapidity, cost-effectiveness, eco-friendliness, and in-field applicability [20]. Electrochemical methods have been developed to characterize the total antioxidant parameters of cognacs and brandies caused by the presence of natural phenolic antioxidants (phenolic acids and aromatic aldehydes) [21,22,23,24]. Electrooxidation of antioxidants on the multi-walled carbon nanotubes (MWCNTs)-modified electrode [22,23] and the polarographic assay based on hydrogen peroxide scavenging [24] are considered. The possibility to evaluate brandy adulteration has been shown using coulometric titration, differential pulse voltammetry, and chronoamperometry on the basis of the total antioxidant parameters [3].

Selective electrochemical determination of individual antioxidants is complicated due to the insufficient selectivity of the electrode response to the structurally related compounds that is one of the most important limitations in the practical application of electrochemical sensors. Sensing layer modification is used to solve this problem. However, cognac and brandy antioxidants as analytes are almost out of the investigation stage. The layer-by-layer combination of MWCNTs and electropolymerized pyrocatechol violet has been reported as a sensing system for the simultaneous quantification of gallic and ellagic acids in cognac and brandy [25]. As for the aromatic aldehydes, the only voltammetric sensor for the simultaneous determination of syringaldehyde and vanillin is based on the glassy carbon electrode (GCE) modified with carbon nanofibers and cetylpyridinium bromide [26]. The analytical characteristics of aromatic aldehydes are not impressive, as the full resolution of the oxidation peaks has not been achieved. The application to real samples has not been performed. Thus, development of voltammetric sensors for the simultaneous quantification of aromatic aldehydes in cognac and brandy is of practical interest.

Electropolymerized coverages are widely studied modifiers of the electrode surface to date. Among various types of monomers, amino groups containing compounds (amino acids [27,28,29,30], aminophenols [31,32,33,34], aminobenzoic acids [35,36,37]) have been shown as effective modifiers of the electrode surface that provide significant improvement of the organic analytes response. Electrodes modified with MWCNTs and oxidized MWCNTs functionalized with 4-aminophenyl phosphonic acid [38] and aminobenzene acids [39] have been reported. Electropolymerized *p*-aminobenzoic acid (*p*-ABA)-based sensors have been reported for the various types of organic analytes, i.e., food azo dyes [40], toxic phenols [41,42], and pharmaceuticals [43,44,45]. MWCNTs have been used as a platform for the electrodeposition of polymers due to the high conductivity and surface area allowing a higher load of polymer [40,45]. The application of *p*-ABA-modified electrodes for the quantification of natural phenolics has not been reported to date and can be a promising approach.

The current work deals with the development of sensitive and selective voltammetric sensor based on the MWCNTs and electropolymerized *p*-aminobenzoic acid (*p*-ABA) for the syringaldehyde and vanillin simultaneous quantification. The conditions of *p*-ABA electropolymerization have been found using voltammetric characteristics of an aromatic aldehydes mixture. The electrooxidation parameters of syringaldehyde and vanillin on poly(*p*-ABA)/MWCNTs/GCE have been studied. The practical applicability of the sensor has been shown on brandy and cognac samples. Comparison to independent chromatographic analysis has been performed.

## 2. Materials and Methods

### 2.1. Reagents

*p*-ABA (99% purity) from Sigma-Aldrich (Steinheim, Germany), vanillin (99%) from Sigma (Steinheim, Germany), and syringaldehyde (98%) from Aldrich (Steinheim, Germany) were used. Their standard solutions (25 mM in distilled water for *p*-ABA and 10 mM in ethanol (restificate) for the aromatic aldehydes) were prepared in 5.0 mL flasks by dissolving the exact weight of the substance. Ascorbic (99%), gallic (99%), and ellagic (95%) acids (Sigma, Steinheim, Germany) were used in the interference study. Their 10 mM (0.86 mM for ellagic acid) standard solutions were prepared in ethanol. The exact dilution was used for the preparation of less concentrated solution before measurements.

MWCNTs (outer diameter 40–60 nm, inner diameter 5–10 nm, and 0.5–500 μm length) from Aldrich (Steinheim, Germany) were used as a platform for the polymeric coverage deposition. A homogeneous 0.5 mg·mL^−1^ suspension of MWCNTs was prepared in 1% sodium dodecylsulfate (Panreac, Barcelona, Spain) using 30 min of sonication in an ultrasonic bath (WiseClean WUC-A03H; DAIHAN Scientific Co., Ltd., Wonju-si, Korea).

Other chemicals were of c.p. grade and were used as received. All experiments were carried out at the laboratory temperature (25 ± 2 °C).

### 2.2. Equipment

The potentiostat/galvanostat Autolab μAutolab Type III (Eco Chemie B.V., Utrecht, The Netherlands) and GPES 4.9 software (Eco Chemie B.V., Utrecht, The Netherlands) were used for the electrochemical measurements. Electrochemical impedance spectroscopy (EIS) was performed on the potentiostat/galvanostat Autolab PGSTAT 302N with the FRA 32M module (Eco Chemie B.V., Utrecht, The Netherlands) and the NOVA 1.10.1.9 software (Eco Chemie B.V., Utrecht, The Netherlands).

A three-electrode system containing a working bare GCE of 3 mm diameter (CH Instruments, Inc., Bee Cave, TX, USA) or a modified GCE (MWCNT_S_/GCE or poly(*p*-ABA)/MWCNT_S_/GCE), a Ag/AgCl reference electrode, and a platinum auxiliary electrode and 10 mL glass electrochemical cell were used in the study.

The pH measurements were carried out using a glassy electrode and “Expert-001” pH meter (Econix-Expert Ltd., Moscow, Russian Federation).

Scanning electron microscopy was performed on the Merlin^TM^ high-resolution field emission scanning electron microscope (Carl Zeiss, Oberkochen, Germany) at the accelerating voltage of 5 kV and emission current of 300 pA.

### 2.3. Procedures

#### 2.3.1. Electrode Surface Modification

The mechanical cleaning of the GCE surface with 0.05 µm of alumina slurry and the following rinse with acetone and distilled water were used prior to the measurements. GCE surface modification was performed using the drop casting method of 4 µL of MWCNTs suspension.

Poly(*p*-ABA) coverage was obtained by potentiodynamic polarization. After achieving a stable supporting electrolyte (Britton–Robinson buffer) curve using five times potential cycling, an aliquot of *p*-ABA solution was inserted in the electrochemical cell and potentiodynamic electrodeposition of the polymer was carried out. *p*-ABA electropolymerization conditions (pH of Britton–Robinson buffer, *p*-ABA concentration, number of cycles, polarization window, and potential scan rate) were optimized on the basis of the voltammetric characteristics of the aromatic aldehydes mixture.

#### 2.3.2. Electrochemical Measurements

Five scans of the supporting electrolyte (Britton–Robinson buffer of various pH) were recorded to achieve a stable blank curve. Then, an aliquot portion of the individual aromatic aldehydes (syringaldehyde or vanillin) solutions or their mixture was inserted in the electrochemical cell (ethanol portion in the cell was reduced to 10% (*v*/*v*)) and cyclic voltammograms were recorded in the range of 0.3–1.2 V at the potential scan rate of 100 mV·s^−1^ or differential pulse voltammograms in the range of 0.5–1.1 V with the potential scan rate of 10 mV·s^−1^. Optimization of pulse parameters was performed on the basis of the syringaldehyde and vanillin mixture response. The oxidation currents in differential pulse voltammetry were calculated using moving average algorithm baseline correction in GPES 4.9 software (Eco Chemie B.V., Utrecht, The Netherlands).

Chronoamperometry was performed at 0.60 V for 25 s using 1.0 mM ferrocyanide ions in 0.1 M KCl.

A 1.0 mM mixture of ferro-/ferricyanide ions in 0.1 M KCl was used as a redox probe in the electrochemical impedance spectroscopy (EIS). The impedance spectra were recorded in the frequency range of 10 kHz–0.04 Hz with an applied sine potential amplitude of 5 mV at a polarization potential of 0.21 V, which was calculated as a half-sum of the redox peaks of ferro-/ferricyanide ions in cyclic voltammetry. The Randles equivalent circuit was applied for the Nyquist plots using NOVA 1.10.1.9 software. The *R*_s_(*R*_et_*Q*) circuit was used for the bare GCE and the *R*_s_(*Q*[*R*_et_*W*]) circuit—for the modified electrodes, where *R*_s_—the electrolyte resistance, *R*_et_—the electron transfer resistance, *Q*—the constant-phase element, and *W*—the Warburg impedance [46].

#### 2.3.3. Real Samples Analysis

Commercial samples of cognac and brandy were studied. Direct analysis was carried out without pretreatment. Britton–Robinson buffer pH 2.0 was added to the electrochemical cell and five blank curves were recorded. Then, an aliquot of the sample was added (40 or 500 µL) and a differential pulse voltammogram from 0.5 to 1.1 V was registered at the pulse amplitude of 50 mV, pulse time of 25 ms, and potential scan rate of 10 mV·s^−1^. Baseline correction was used for the calculations of the oxidation currents.

#### 2.3.4. Data Treatment

All electrochemical measurements were performed in five replicates (three replicates for chromatography). Statistical treatment of the data was carried out at *α* = 0.05. Experimental data were presented as the average value ± coverage interval. The random error was reflected by the relative standard deviation (RSD). *F*- and *t*-tests were applied for the validation of the developed approach.

The detection limits were calculated as 3SD_a_/*b*, where SD_a_ was the standard deviation of the calibration graph intercept and *b*—the calibration graph slope.

OriginPro 8.1 software (OriginLab, Northampton, MA, USA) was used for the regression and statistical analysis.

## 3. Results and Discussion

### 3.1. Voltammetric Characteristics of Aromatic Aldehydes at the Bare GCE and MWCNTs-Modified Electrode

**Figure 1:**
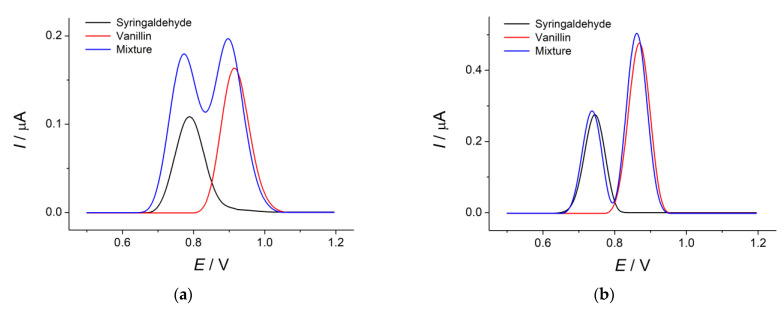
Baseline-corrected differential pulse voltammograms of 10 µM syringaldehyde, vanillin, and their mixture: (**a**) at the bare GCE; (**b**) at the MWCNTs/GCE. The supporting electrolyte is Britton–Robinson buffer pH 2.0. Pulse amplitude is 50 mV, pulse time is 50 ms, and potential scan rate is 10 mV·s^−1^.

Syringaldehyde and vanillin are oxidized at the bare GCE in Britton–Robinson buffer pH 2.0. The oxidation peaks at 0.782 and 0.913 V are observed for syringaldehyde and vanillin, respectively, on differential pulse voltammograms (Figure 1a). Peak potential separation is insufficient and makes the analytes peak resolution impossible in simultaneous presence. The oxidation peaks become closer to each other and partially overlap even in the differential pulse mode (Figure 1a).

As known [47,48], the electrode surface modification with MWCNTs improves the voltammetric response of natural phenolics including vanillin. Therefore, the voltammetric behavior of syringaldehyde and vanillin at the MWCNTs/GCE is investigated. Oxidation peaks at 0.742 and 0.873 V are registered for syringaldehyde and vanillin, respectively (Figure 1b), i.e., a shift in the oxidation potentials to less positive values on 40 mV vs. GCE is achieved for both aldehydes. This effect confirms the increase in the electron transfer rate at the MWCNTs/GCE [49]. The oxidation currents are 2.2- and 2.6-fold increased for syringaldehyde and vanillin, respectively, which is caused by the larger effective surface area of the modified electrode. The peak potential separation of 131 mV is similar to that at the bare GCE. Nevertheless, two almost fully separated peaks at 0.742 and 0.863 mV are observed in the voltammograms of the aldehyde’s mixture (Figure 1b). However, the higher sensitivity of the response and better resolution of the oxidation peaks is required for the simultaneous determination of the aldehydes in real samples.

A poly(*p*-ABA)-modified electrode is developed for these purposes.

### 3.2. Electropolymerization of p-ABA at the MWCNTs-Modified Electrode

**Figure 2:**
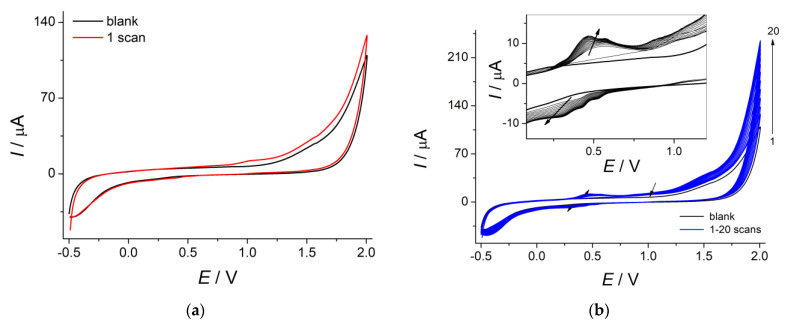
Electropolymerization of 100 µM *p*-ABA at the MWCNTs/GCE in Britton–Robinson buffer pH 2.0: (**a**) First cycle of electropolymerization; (**b**) Twenty cycles of electropolymerization. The inset is the enlarged scale of voltammograms in the potential range of 0.1–1.2 V. Potential scan rate is 100 mV·s^−1^.

**Scheme 1:**
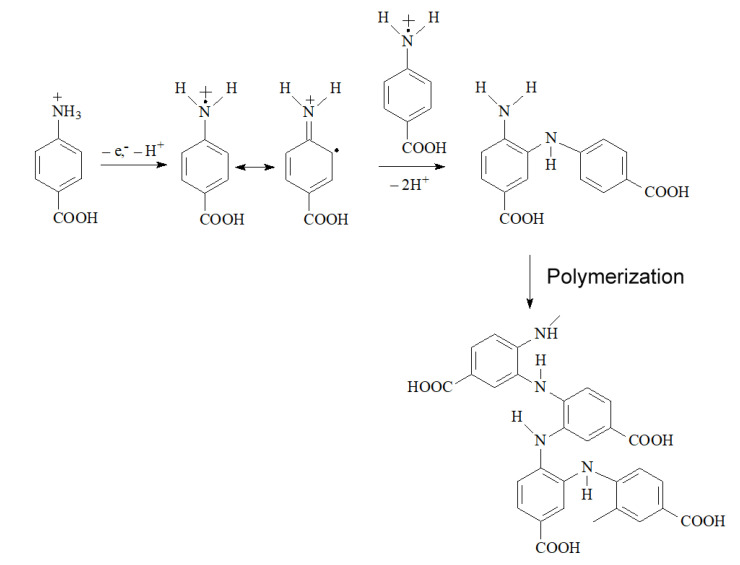
Electropolymerization of *p*-ABA in acidic medium.

There is an irreversible oxidation step at 1.0 V in the cyclic voltammograms of *p*-ABA in Britton–Robinson buffer pH 2.0 (Figure 2a). This step corresponds to cation radical formation (Scheme 1). Oxidation currents are decreased as the number of cycles increase up to full disappearance, which agree well with the report [50]. On the other hand, the quasi-reversible redox pair at 450–500 mV appears on the second and following scans (Figure 2b). This pair transforms into two quasi-reversible redox pairs at 0.44/0.42 and 0.59/0.54 V after the fifth cycle (Figure 2b insert). Corresponding currents are increased with the increase in the number of cycles that confirms the formation of conductive polymeric coverage [36,50,51,52] according to Scheme 1.

The electropolymerization conditions (supporting electrolyte pH, *p*-ABA concentration, polarization window, and potential scan rate) are optimized to obtain the best response of the syringaldehyde and vanillin mixture. A 120 mV peak potential separation is not affected by electropolymerization conditions. Syringaldehyde and vanillin oxidation currents change significantly and are used for the optimization of *p*-ABA electropolymerization.

**Figure 3:**
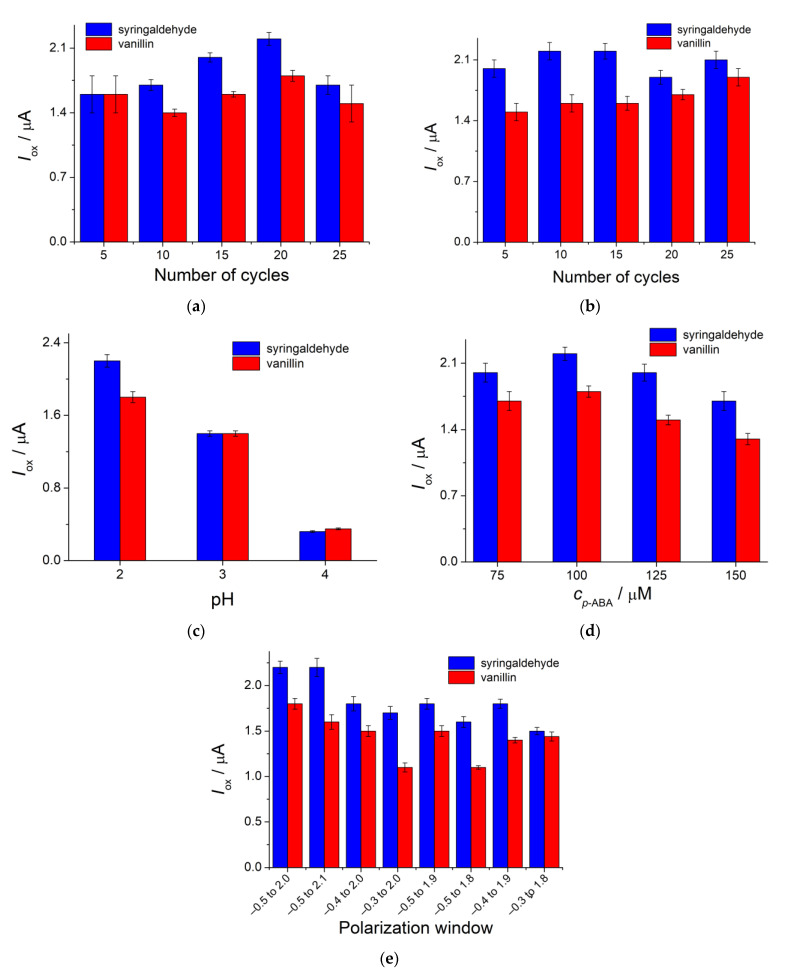
Changes in the oxidation currents of 10 µM mixture of syringaldehyde and vanillin at the polymer-modified electrode depending on the: (**a**) number of cycles at υ = 100 mV·s^−1^; (**b**) number of cycles at υ = 150 mV·s^−1^; (**c**) supporting electrolyte pH; (**d**) monomer concentration; (**e**) polarization window used for the poly(*p*-ABA) layer electrodeposition. The response of aromatic aldehydes is recorded in Britton–Robinson buffer pH 2.0 using cyclic voltammetry at a potential scan rate of 100 mV·s^−1^.

The variation in the number of cycles at different potential scan rates (Figure 3a,b) shows that the maximal oxidation currents of the aldehydes and the best current ratio are achieved for 20 potential cycles at a scan rate of 100 mV·s^−1^.

The changes in the oxidation currents of syringaldehyde and vanillin in the mixture depending on the supporting electrolyte pH (Britton–Robinson buffer) used for the *p*-ABA electropolymerization is evaluated (Figure 3c). The increase in pH leads to a significant decrease in the oxidation currents and their ratio for both aldehydes. Thus, Britton–Robinson buffer pH 2.0 is chosen as the medium for *p*-ABA electropolymerization.

The concentration of the monomer and polarization window show a notable influence on the syringaldehyde and vanillin oxidation currents (Figure 3d,e). Varying the monomer concentration from 75 to 150 µM, significant changes in the oxidation currents of aldehydes are obtained while the currents ratio remains approximately the same. Maximal currents are achieved for 100 µM *p*-ABA (Figure 3d). The polarization window also affects the syringaldehyde and vanillin response (Figure 3e). The highest oxidation currents are observed on the poly(*p*-ABA) layer formed in the polarization window from −0.5 to 2.0 V. The narrowing and expansion of the potential range leads to a decrease in the oxidation currents of both aldehydes, which is probably caused by the completeness of the *p*-ABA electropolymerization at the electrode surface.

The optimized conditions for *p*-ABA electropolymerization are found as 100 µM monomer in Britton-Robinson buffer pH 2.0, twenty cycles in the polarization window from −0.5 to 2.0 V, and a potential scan rate of 100 mV·s^−1^.

#### 3.2.1. Electrochemical Behavior of Poly(p-ABA)-Modified Electrode

Poly(*p*-ABA)/MWCNTs/GCE demonstrates electroactivity in the supporting electrolyte that agrees well with the report [36,50,51,52]. Two quasi-reversible redox pairs at 0.44/0.42 and 0.59/0.54 V are observed in the cyclic voltammograms in Britton–Robinson buffer pH 2.0 (Figure S1). A similar behavior has been reported in 0.1 M H_2_SO_4_ [52]. The capacitive currents for the polymer-modified electrode are significantly increased compared to MWCNTs/GCE, as Figure S1 shows.

Both redox pair potentials are shifted to less values with a pH increase (Figure S2). Furthermore, the second redox pair disappears at pH 4.0. The slope of 60 mV per pH unit for the first redox pair indicates an equal number of electrons and protons participating in the electrode reaction. Redox currents are significantly decreased as the pH increases. There are no redox signals of poly(*p*-ABA)/MWCNTs/GCE in Britton–Robinson buffer pH 7.0.

### 3.3. Characterization of the Electrodes Morphology and Electrochemical Properties

#### 3.3.1. Scanning Electron Microscopy (SEM)

**Figure 4:**
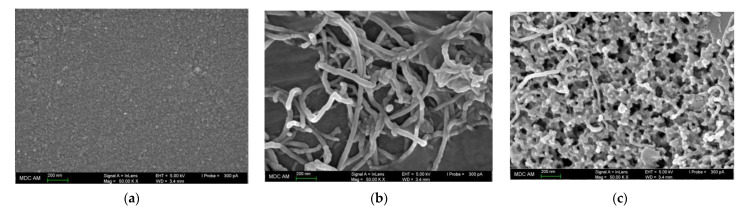
SEM images of: (**a**) bare GCE; (**b**) MWCNTs/GCE; (**c**) Poly(*p*-ABA)/MWCNTs/GCE.

SEM characterization of the electrodes’ morphology is performed (Figure 4).

Successful immobilization of the MWCNTs and polymeric coverage is confirmed. A network structure formed by MWCNTs of 40–70 nm thickness and their aggregates included in the surfactant film is obtained for the MWCNTs/GCE (Figure 4b). Polymeric coverage exhibits evenly distributed odd-shaped structures (Figure 4c). This leads to the formation of the porous surface of poly(*p*-ABA)/MWCNTs/GCE. The pores size is varied in the range of 50–200 nm. The modified electrodes surface is of high roughness, i.e., the area of the electrodes is increased vs. bare GCE.

#### 3.3.2. Cyclic Voltammetry and EIS

**Figure 5:**
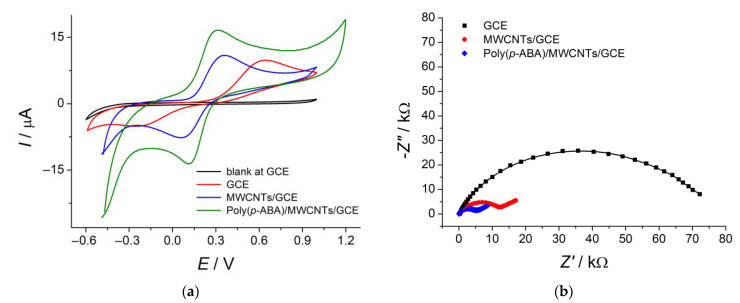
(**a**) Cyclic voltammograms of 1.0 mM ferrocyanide ions in 0.1 M KCl at the bare GCE, MWCNTs/GCE, and poly(*p*-ABA)/MWCNTs/GCE. The potential scan rate is 100 mV·s^−1^; (**b**) Nyquist plot (experimental (points) and fitted (lines)) for bare GCE, MWCNTs/GCE, and poly(*p*-ABA)/MWCNTs/GCE in the presence of 1.0 mM mixture of ferro-/ferricyanide ions in 0.1 M KCl. Polarization potential is 0.21 V, frequency range is 10 kHz—0.04 Hz, amplitude is 5 mV.

Changes in the surface structure affect the electrochemical properties of the modified electrodes. The electroactive surface area is evaluated using cyclic voltammetry of 1.0 mM ferrocyanide ions (Figure 5a).

An irreversible oxidation of the redox probe is observed at the bare GCE, which is in line with the report in [25,40]. Therefore, chronoamperometry at 0.60 V is used for the estimation of GCE electroactive surface area (Figure S3). Electrooxidation of ferrocyanide ions at the modified electrodes proceeds reversibly, allowing application of the Randles–Ševčík equation for the electroactive surface area calculation. The data obtained are presented in Table 1.

The poly(*p*-ABA)-modified electrode shows a statistically significantly higher electroactive surface area compared to the bare GCE and MWCNTs/GCE (10.9- and 1.2-fold, respectively), which agree well with SEM data. Thus, higher oxidation currents of the aromatic aldehydes on the poly(*p*-ABA)/MWCNTs/GCE are caused by the increase in the electroactive surface area of the electrode.

Electron transfer properties are characterized by EIS using a mixture of ferri/ferrocyanide ions as a redox probe. The corresponding impedance spectra are presented as Nyquist plots in Figure 5b. The polymer-modified electrode demonstrates a lower semicircle diameter in the high-frequency region, which means the lowest electron transfer resistance. EIS data agree with cyclic voltammetry. Impedance spectra fitting results based on the application of Randles’ equivalent circuit (Table 1) confirm the increase in the electron transfer rate for the modified electrodes. A 2.06-fold increase in the constant phase element for the polymer-modified electrode vs. MWCNTs/GCE is due to the higher porosity of the electrodes surface as well as its higher conductivity since the polymeric coverage is conductive. A 1.74-fold higher Warburg impedance also indicates conductivity of the polymeric film.

Thus, poly(*p*-ABA) coverage can be considered as an effective electrode surface modifier useful in the organic electroanalysis.

### 3.4. Comparison of the Voltammetric Characteristics of Aromatic Aldehydes at the MWCNTs- and Polymer-Modified Electrodes

**Figure 6:**
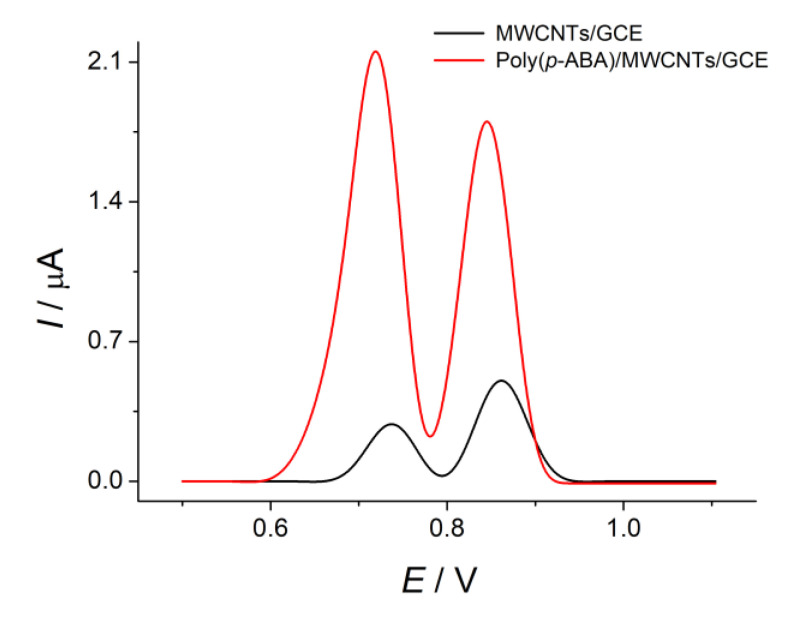
Baseline-corrected differential pulse voltammograms of 10 µM mixture of syringaldehyde and vanillin at the MWCNTs/GCE and poly(*p*-ABA)/MWCNTs/GCE in Britton–Robinson buffer pH 2.0. Pulse amplitude is 50 mV, pulse time is 50 ms, and potential scan rate is 10 mV·s^−1^.

The voltammetric characteristics of the syringaldehyde and vanillin mixture at the MWCNTs- and poly(*p*-ABA)-modified electrodes are compared. Similar to MWCNTs/GCE, well-defined oxidation peaks of syringaldehyde and vanillin are observed at the poly(*p*-ABA)/MWCNTs/GCE (Figure 6).

The corresponding voltammetric characteristics are summarized in Table 2.

Oxidation potentials are shifted to the less positive values for 20 mV for both aldehydes. Oxidation currents are 8.0- and 3.6-fold increased for syringaldehyde and vanillin, respectively. Furthermore, the aldehydes currents ratio is changed at the polymer-modified electrode, i.e., the syringaldehyde response becomes higher than that for vanillin. The data obtained confirm the effectivity of the polymeric coverage as a sensing layer.

### 3.5. Electrooxidation of Aromatic Aldehydes at the Poly(p-ABA)/MWCNTs/GCE

Syringaldehyde and vanillin electrooxidation at the poly(*p*-ABA)-modified electrode is studied using cyclic voltammetry.

#### 3.5.1. Effect of Supporting Electrolyte pH

**Figure 7:**
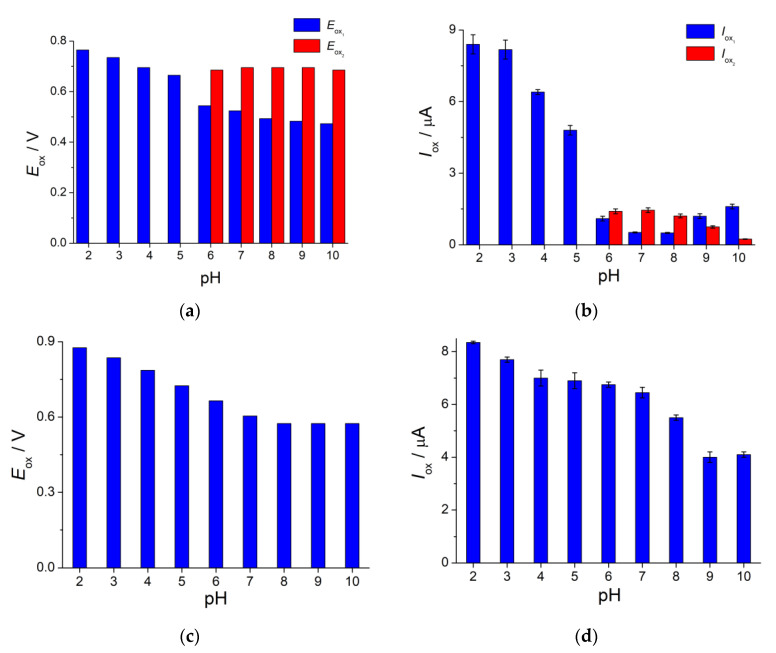
Voltammetric characteristics of 100 µM aromatic aldehydes at the poly(*p*-ABA)-modified electrode depending on the Britton–Robinson buffer pH: (**a**) Oxidation potentials of syringaldehyde; (**b**) Oxidation currents of syringaldehyde; (**c**) Oxidation potentials of vanillin; (**d**) Oxidation currents of vanillin.

The Britton–Robinson buffer pH is varied in the range of 2.0–10.0 (Figure 7).

Oxidation potentials of both aldehydes decrease with the pH increase (Figure 7a,c) indicating proton participation in the electrode reaction. The absence of cathodic steps confirms the irreversibility of the aldehydes oxidation in the whole pH range. A split of the oxidation step and a redistribution of the oxidation currents are observed for the syringaldehyde at pH 6.0 and above (Figure 7a,b). pH-independent oxidation is observed in the basic medium, which agrees well with the dissociation constants (pKas are 7.09 for syringaldehyde [53] and 7.36 for vanillin [54]). Oxidation currents are decreased with increasing Britton–Robinson buffer pH that is caused by partial oxidation of the aldehydes with air oxygen, especially in neutral and basic medium that is typical for the phenolics [55]. Britton–Robinson buffer pH 2.0 is chosen for further investigation as providing the highest oxidation currents of both aldehydes.

#### 3.5.2. Effect of Potential Scan Rate

**Figure 8:**
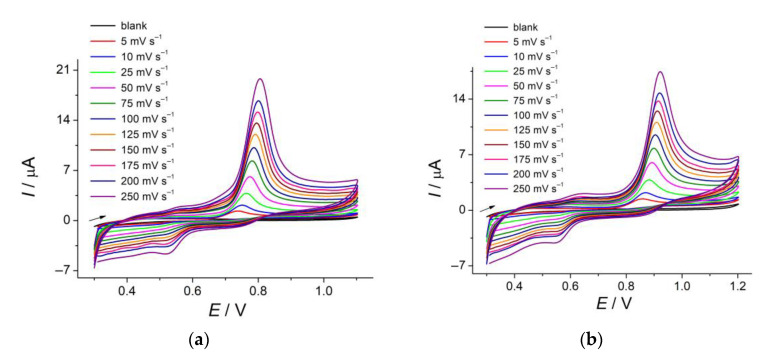
Cyclic voltammograms of aromatic aldehydes at the poly(*p*-ABA)-modified electrode in the Britton–Robinson buffer pH 2.0 at various potential scan rates: (**a**) 100 µM of syringaldehyde; (**b**) 100 µM of vanillin.

The electrooxidation parameters of aromatic aldehydes are evaluated using cyclic voltammetry at the various potential scan rates. There are well-defined oxidation peaks of syringaldehyde and vanillin in the whole range (5–250 mV·s^−1^) of potential scan rates studied (Figure 8). The oxidation potentials of aromatic aldehydes are anodically shifted with the increase in potential scan rate, indicating irreversibility of the electrooxidation that is also confirmed by the absence of cathodic steps on the voltammograms at low potential scan rates. The quasi-reversible redox pair belongs to the polymeric coverage (see Section 3.2.1). The oxidation currents are increased with the potential scan rate growth. The parameters of the corresponding linear plots are summarized in Table 3.

The linear plots of aldehydes oxidation currents vs. square root of the potential scan rate suggest that electrooxidation is a diffusion-controlled process. Nevertheless, the slopes of the plots ln*I*_ox_ vs. lnυ are close to 0.7, which means mixed control, i.e., both diffusion and surface control are realized. Such behavior has been reported for vanillin at polymer-modified electrodes [56,57,58] and often takes place in electrochemistry. Diffusion control is dominant at low scan rates, while the impact of surface processes appears at the higher scan rates. The anodic transfer coefficients are calculated from the Tafel plots [59] at 5 mV·s^−1^ as 0.51 and 0.45 for syringaldehyde and vanillin, respectively.

The slope of the plot *E* = f(lnυ) is used for the calculation of the number of electrons according to Equation (1) [59]

slope = *RT*/2α_a_*nF*, (1)

where *R—*universal gas constant (J·mol^−1^·K^−1^), *T*—temperature (K), α_a_—the anodic transfer coefficient, *n*—the number of electrons participating in oxidation, *F*—the Faraday constant (C·mol^−1^). Electrooxidation of syringaldehyde and vanillin proceeds with the participation of 1.8 and 1.7 electrons, respectively.

**Scheme 2:**
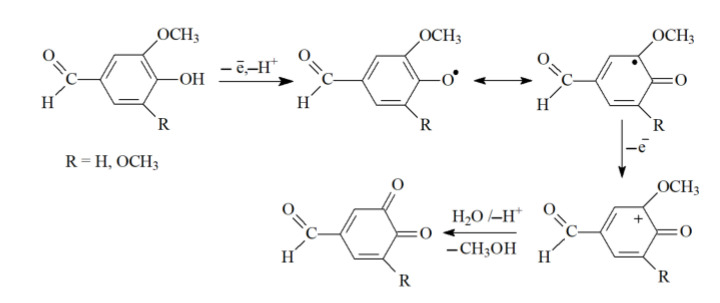
Electrooxidation of aromatic aldehydes.

Thus, electrooxidation of aromatic aldehydes involves two electrons and protons and corresponds to the formation of *o*-quinones (Scheme 2) that agree well with the report in [26,60,61,62,63].

### 3.6. Simultaneous Quantification of Aromatic Aldehydes

Syringaldehyde and vanillin quantification is carried out in differential pulse mode, which is a useful tool for the irreversible redox systems and provides high sensitivity of target analytes response.

The effect of pulse parameters on the voltammetric characteristics of aromatic aldehydes is studied. The oxidation potentials of both aldehydes in the mixture are shifted at 10 mV to less-positive values with the increase in pulse amplitude and time. The peak potential separation is kept the same. The oxidation currents of aromatic aldehydes are changed significantly (Figure S4). The increase in the pulse amplitude from 25 to 50 mV leads to a statistically significant growth of the oxidation currents. A pulse amplitude of 75 mV cannot be applied, due to the worse resolution of the syringaldehyde and vanillin oxidation peaks. The oxidation currents of aldehydes are significantly decreased as pulse time increases. The effect is more pronounced for syringaldehyde, although similar changes are observed for vanillin at 75 and 100 ms (Figure S4b). The best voltammetric characteristics are obtained for a pulse amplitude of 50 mV and a pulse time of 25 ms.

**Figure 9:**
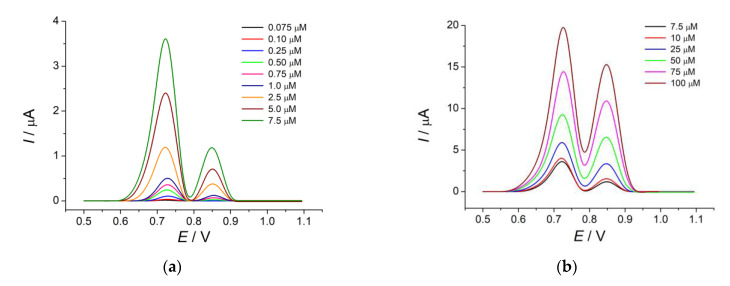
Baseline-corrected differential pulse voltammograms of the aromatic aldehydes equimolar mixtures of various concentrations at the poly(*p*-ABA)-modified electrode in the Britton–Robinson buffer pH 2.0: (**a**) concentration range of 0.075–7.5 µM; (**b**) concentration range of 7.5–100 µM. Pulse amplitude is 50 mV, pulse time is 25 ms, potential scan rate is 10 mV·s^−1^.

Aromatic aldehydes mixtures give well-defined oxidation peaks at 0.72 and 0.84 V on the voltammograms (Figure 9).

Syringaldehyde oxidation currents are significantly higher than for the vanillin, especially in the low-concentration range. The oxidation currents of both aldehydes are proportionally increased with the concentration growth. The analytical characteristics achieved and calibration graphs parameters are shown in Table 4. The sensitivity of vanillin determination is threefold lower than for the syringaldehyde in the first linear range.

The analytical characteristics obtained are significantly improved compared to those reported for the simultaneous determination of syringaldehyde and vanillin using voltammetry [26] and other methods [8,9,10,11,12,13,15,16,17] (Table 5), as well as for the individual determination of the aldehydes [48,63,64,65,66,67,68,69,70] (Table S1).

Investigation of non-equimolar mixtures of syringaldehyde and vanillin (Figure S5) shows independent electrooxidation in the first linear range, which is an important advantage of the approach developed. The oxidation currents are the same as for the equimolar mixtures. Therefore, the calibration graph for equimolar mixtures can be used for the quantification of aldehydes independently of their concentrations ratio that significantly simplifies real samples analysis. The sample dilution can be applied in the case of high concentrations of the syringaldehyde and vanillin.

The method developed is tested for the accuracy and reproducibility using model mixtures of aromatic aldehydes (Table 6).

Five levels from the entire linear dynamic range are checked. The recovery values of 98–102% indicate high accuracy of the sensor developed. The sensor surface is fully renewed by mechanical treatment after each measurement due to its fouling with the aldehydes oxidation products. Therefore, the RSD in Table 6 reflects the reproducibility of the analytical signal. RSD values do not exceed 3.4%, confirming high reproducibility of the sensor response to syringaldehyde and vanillin. Furthermore, the RSD obtained indicates the absence of random errors of determination.

#### Interference Study

The interference study is carried out with a 1.0 µM mixture of aromatic aldehydes. Typical and specific interferences are considered. Inorganic ions (K^+^, Mg^2+^, Ca^2+^, NO_3_^−^, Cl^−^, and SO_4_^2−^) are electrochemically “silent” in the potential range used and even a 1000-fold excess does not show an effect on the oxidation peaks of syringaldehyde and vanillin. Glucose, rhamnose, and sucrose do not oxidize under conditions of the experiments. These saccharides up to 100-fold excess do not affect the response of aromatic aldehydes. Ascorbic acid is oxidized at the poly(*p*-ABA)/MWCNTs/GCE that leads to the insignificant increase in the polymeric coverage oxidation currents in the potential range of 0.2–0.4 V. Nevertheless, there is no interference effect of ascorbic acid even at 100-fold excess. Phenolic acids, namely gallic and ellagic acids, being the major phenolics of brandy and cognac, are electrochemically active at the electrode developed. The oxidation potentials of 0.58 and 0.69 V for gallic and ellagic acids, respectively, are found. The peak potential separation of ellagic acid and syringaldehyde is insufficient to obtain well-resolved oxidation peaks. Nevertheless, the sensitivity of the sensor response to gallic and ellagic acids is significantly less compared to aromatic aldehydes. A 10-fold excess of gallic acid and ellagic acid in quantities less than 1.0 µM does not show an interference effect. Thus, the developed sensor shows sufficient selectivity for syringaldehyde and vanillin that allows its application in real samples analysis.

### 3.7. Real Samples Analysis

Practical applicability of the developed sensor is shown on the cognac and brandy samples of various denominations and origins.

Taking into account the ratio of syringaldehyde to vanillin in cognac and brandy, the volume of the sample to be used for the analysis is found first. A significantly lower concentration of vanillin in combination with a lower sensitivity of vanillin response requires high aliquots of the sample. On the other hand, the interference effect of ellagic acid on the determination of syringaldehyde occurs in this case. Therefore, various aliquots of the sample have to be used for the determination of syringaldehyde and vanillin. As confirmed by experiment, 40 and 500 µL of the sample must be used for the quantification of syringaldehyde and vanillin, respectively.

Well-defined oxidation peaks at 0.72 and 0.84 V are observed in the voltammograms of cognac and brandy corresponding to the oxidation of syringaldehyde and vanillin, respectively (Figure S6), which is also confirmed by the standard addition method (Table 7). A proportional increase in the oxidation currents at the corresponding potential is registered after addition of the analytes to the samples. The recovery values (95.7–101%) indicate the absence of matrix effects and high accuracy of the aromatic aldehydes quantification.

The quantification of syringaldehyde and vanillin in cognac and brandy samples is summarized in Table 8. The RSD values do not exceed 7% for the brandy with low contents of aromatic aldehydes and 1% for other samples. The sensor developed is compared to HPLC with UV-detection [10]. The data obtained by both methods agree well. The results of the *t*- and *F*-tests are less than critical values, which indicates the absence of systematic errors in the determination of aldehydes and the similar accuracy of both methods.

As known [4,5,16], an increased content of vanillin can indicate adulteration if used for the mimeting of the beverage organoleptic properties. Thus, the method developed can be applied for the primary control of the cognac and brandy quality prior to the detailed chromatographic analysis.

## Figures and Tables

**Table 1:** Electroactive surface area and fitted parameters of the electrochemical impedance for the electrodes (*n* = 5; *p* = 0.95).

Electrode	*A* (mm^2^)	*R*_s_ (Ω)	*R*_et_ (kΩ)	*Q* (µΩ^−1^)	*n*	*W* (µΩ^−1^)	χ^2^
GCE	8.2 ± 0.3	245 ± 5	72 ± 3	3.7 ± 0.2	0.789	-	0.028
MWCNTs/GCE	75 ± 3	199 ± 3	12.1 ± 0.9	3.6 ± 0.1	0.775	230 ± 5	0.015
Poly(*p*-ABA)/MWCNTs/GCE	89 ± 4	100 ± 1	4.9 ± 0.3	7.4 ± 0.2	0.875	401 ± 4	0.012

**Table 2:** Voltammetric characteristics of 10 µM mixture of aromatic aldehydes at the MWCNTs- and poly(*p*-ABA)-modified electrodes in Britton–Robinson buffer pH 2.0 (*n* = 5; *P* = 0.95).

Electrode	Syringaldehyde		Vanillin	
	*E*_ox_ (V)	*I*_ox_ (µA)	*E*_ox_ (V)	*I*_ox_ (µA)
MWCNTs/GCE	0.742	0.28 ± 0.01	0.863	0.50 ± 0.01
poly(*p*-ABA)/MWCNTs/GCE	0.722	2.21 ± 0.07	0.842	1.82 ± 0.06

**Table 3:** Effect of the potential scan rate on the voltammetric characteristics of aromatic aldehydes at the poly(*p*-ABA)-modified electrode in Britton–Robinson buffer pH 2.0.

Aromatic Aldehyde	Linear Regression Parameters		
	*a* ± SD	*b* ± SD	*R* ^2^
*I* (µA) = *a* + *b*υ^1/2^ (mV·s^−1^)			
Syringaldehyde	−2.87 ± 0.44	1.17 ± 0.04	0.9893
Vanillin	−1.74 ± 0.25	0.94 ± 0.02	0.9931
ln*I* (µA) = *a* + *b*lnυ (V·s^−1^)			
Syringaldehyde	3.78 ± 0.01	0.718 ± 0.004	0.9997
Vanillin	3.59 ± 0.02	0.699 ± 0.005	0.9994
*E* (V) = *a* + *b*lnυ (V·s^−1^)			
Syringaldehyde	0.815 ± 0.002	0.0137 ± 0.0006	0.9941
Vanillin	0.946 ± 0.003	0.0176 ± 0.0009	0.9718

**Table 4:** Analytical characteristics and parameters of calibration graphs for aromatic aldehydes.

Analyte	Detection Limit (µM)	Linear Dynamic Range (µM)	*I* = *a* + *bc*		
			*a* ± SD (µA)	(*b* ± SD) × 10^−4^ (µA·M^−1^)	*R* ^2^
Syringaldehyde	0.018	0.075–7.50	−0.014 ± 0.003	48.80 ± 0.09	0.9999
		7.50–100	2.74 ± 0.05	12.94 ± 0.09	0.9998
Vanillin	0.19	0.50–7.50	−0.04 ± 0.01	15.61 ± 0.36	0.9973
		7.50–100	0.43 ± 0.11	11.18 ± 0.18	0.9990

**Table 5:** Analytical characteristics of the simultaneous determination of aromatic aldehydes.

Method	Analyte	Detection Limit (µM)	Linear Dynamic Range (µM)	Ref
HPLC-UV	Syringaldehyde	3.07	0.27–1255	[8]
	Vanillin	1.05	0.59–1502	
HPLC-DAD ^1^	Syringaldehyde	0.55	27.4–2189	[9]
	Vanillin	1.31	32.9–2629	
	Syringaldehyde	0.16	0.16–220	[10]
	Vanillin	0.26	0.99–197	
	Syringaldehyde	0.19	0.69–41.2	[11]
	Vanillin	0.079	0.33–49.3	
Ultra-HPLC-UV	Syringaldehyde	0.37	0.97–97.4	[12]
	Vanillin	0.37	1.12–113	
Ultra-HPLC- MS/MS	Syringaldehyde	4.0 × 10^−8^	0.1–100	[13]
	Vanillin	4.1 × 10^−8^	0.1–100	
	Syringaldehyde	0.07	0.27–54.9	[15]
	Vanillin	0.076	0.33–65.7	
Capillary electrophoresis	Syringaldehyde	0.88	2.74–110	[16]
	Vanillin	0.92	3.29–131	
	Syringaldehyde	0.78	1.65–313	[17]
	Vanillin	1.81	1.97–375	
DPV ^2^ using cetylpyridinium bromide/carbon nanofibers/GCE	Syringaldehyde	0.53	2.5–30	[26]
	Vanillin	1.17	5.0–40	
DPV using poly(*p*-ABA)/MWCNTs/GCE	Syringaldehyde	0.018	0.075–7.5; 7.5–100	This work
	Vanillin	0.19	0.50–7.5; 7.5–100	

^1^ Diode-array detection. ^2^ Differential pulse voltammetry.

**Table 6:** Simultaneous quantification of aromatic aldehydes in model mixtures at the poly(*p*-ABA)/MWCNTs/GCE in Britton–Robinson buffer pH 2.0 (*n* = 5; *p* = 0.95).

Aldehyde	Added (µg)	Found (µg)	RSD (%)	Recovery (%)
Syringaldehyde	0.068	0.066 ± 0.001	1.3	97.85 ± 2.01
	0.68	0.68 ± 0.03	3.4	100.00 ± 4.20
	6.8	6.80 ± 0.05	0.59	100.00 ± 0.73
	23	23.04 ± 0.0.68	2.4	100.17 ± 3.04
	91	90.86 ± 0.44	0.39	99.85 ± 0.44
Vanillin	0.38	0.381 ± 0.008	1.7	100.26 ± 2.01
	1.9	1.93 ± 0.06	2.3	102.02 ± 2.95
	5.7	5.70 ± 0.06	0.79	100.00 ± 1.01
	38	37.90 ± 0.37	0.79	99.73 ± 0.91
	76	75.60 ± 0.87	0.93	99.47 ± 0.79

**Table 7:** Recovery of syringaldehyde and vanillin in brandy and cognac samples (*n* = 5; *p* = 0.95).

Sample	Analyte	Spiked (µg)	Found (µg)	RSD (%)	Recovery (%)
Brandy	Syringaldehyde	0	0.150 ± 0.006	3.0	
		0.080	0.225 ± 0.006	2.2	97.82 ± 2.61
		0.15	0.30 ± 0.02	3.3	100.00 ± 3.01
	Vanillin	0	0.77 ± 0.05	2.6	
		0.14	0.90 ± 0.03	2.0	98.90 ± 2.98
		0.27	1.04 ± 0.02	1.5	100.00 ± 1.96
Cognac	Syringaldehyde	0	0.40 ± 0.02	2.5	
		0.20	0.61 ± 0.03	4.1	101.67 ± 3.85
		0.40	0.80 ± 0.02	0.95	100.00 ± 1.05
	Vanillin	0	0.76 ± 0.04	2.0	
		0.13	0.90 ± 0.03	2.4	101.12 ± 3.11
		0.25	1.01 ± 0.01	1.1	100.00 ± 0.99

**Table 8:** Quantification of syringaldehyde and vanillin in brandy and cognac samples (*p* = 0.95).

Sample	Analyte	Aromatic Aldehydes Contents (mg·L^−1^)				*t*-Test ^1^	*F*-Test ^2^
		Voltammetry	RSD (%)	Chromatography	RSD (%)		
Brandy 5-Star	Syringaldehyde	3.71 ± 0.02	0.54	3.72 ± 0.01	0.16	0.548	11.89
	Vanillin	1.84 ± 0.03	1.1	1.86 ± 0.01	0.31	2.19	11.89
Brandy VS	Syringaldehyde	0.35 ± 0.03	7.3	0.33 ± 0.06	7.7	1.05	1.00
	Vanillin	0.31 ± 0.03	6.8	0.31 ± 0.05	6.5	0.0100	1.10
Cognac XO	Syringaldehyde	8.7 ± 0.2	1.4	8.68 ± 0.07	0.35	0.0530	17.36
	Vanillin	4.45 ± 0.04	0. 08	4.48 ± 0.02	0.22	0.791	12.25

^1^ *t*_crit_ = 2.45 at α = 0.05 and *f* = 6. ^2^ *F*_crit_ = 19.25 at α = 0.05 and *f*_1_ = 4 and *f*_2_ = 2.

## Data Availability

The data presented in this study are available in the electronic Supplementary Materials.

## Supplementary Materials

The following supporting information can be downloaded at: https://www.mdpi.com/article/10.3390/s23042348/s1, Figure S1: Cyclic voltammograms of MWCNTs/GCE and poly(*p*-ABA)/MWCNTs/GCE in Britton–Robinson buffer pH 2.0; Figure S2: Cyclic voltammograms of poly(*p*-ABA)/MWCNTs/GCE in Britton–Robinson buffer at various pH. Potential scan rate is 100 mV·s^−1^; Figure S3: (**a**) Chronoamperometric curves of 1.0–3.0 mM ferrocyanide ions in 0.1 M KCl at the bare GCE at 0.60 V; (**b**) Plot of *I* vs. *t* ^–½^ based on the chronoamperometric data; Figure S4: Effect of pulse parameters on the oxidation currents of 10 µM mixture of aromatic aldehydes at the poly(*p*-ABA)/MWCNTs/GCE in Britton–Robinson buffer pH 2.0: (**a**) syringaldehyde; (**b**) vanillin; Table S1: Figures of merit of electrochemical sensors for the simultaneous and individual determination of aromatic aldehydes; Figure S5. Baseline-corrected differential pulse voltammograms of non-equimolar mixtures of aromatic aldehydes at the poly(*p*-ABA)-modified electrode in the Britton–Robinson buffer pH 2.0: (**a**) 0.75–5.0 µM of syringaldehyde in the presence of 7.5 µM vanillin; (**b**) 0.75–5.0 µM of vanillin in the presence of 7.5 µM syringaldehyde. Pulse amplitude is 50 mV, pulse time is 25 ms, potential scan rate is 10 mV·s^−1^; Figure S6: Typical baseline-corrected differential pulse voltammograms of brandy at the poly(*p*-ABA)/MWCNTs/GCE in Britton–Robinson buffer pH 2.0: (**a**) 40 µL of brandy with various additions of syringaldehyde; (**b**) 500 µL of brandy with various additions of vanillin. Pulse amplitude is 50 mV, pulse time is 25 ms, potential scan rate is 10 mV·s^−1^.
