# Supplementary material for: Voltammetric Sensor Based on the Poly(p-aminobenzoic Acid) for the Simultaneous Quantification of Aromatic Aldehydes as Markers of Cognac and Brandy Quality
Source: Sensors (Basel). 2023 Feb 20;23(4):2348. doi: 10.3390/s23042348 (PMC9960838; doi:10.3390/s23042348)
Supplement: Supplementary file 1 [file sensors-23-02348-s001.zip › sensors-2164279-supplementary.pdf]

Supplementary Materials

Voltammetric Sensor Based on the Poly(*p*-Aminobenzoic Acid) for the Simultaneous Quantification of Aromatic Aldehydes as a Markers of Cognac and Brandy Quality

Guzel Ziyatdinova *, Tatyana Antonova and Rustam Davletshin

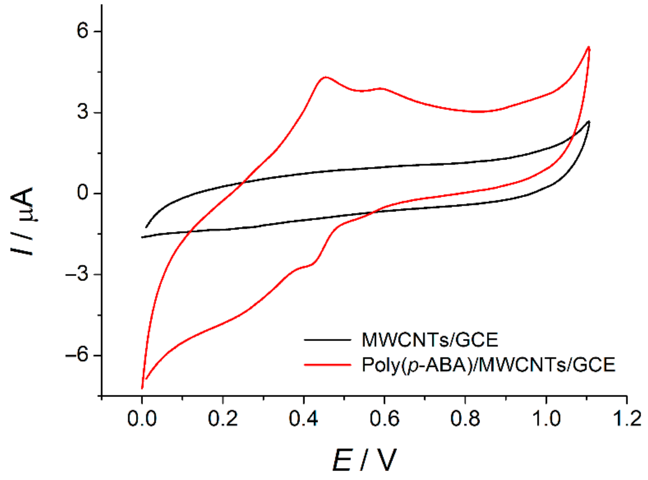

Figure S1. Cyclic voltammograms of Britton-Robinson buffer pH 2.0 at MWCNTs/GCE and poly(*p*-ABA)/MWCNTs/GCE. Potential scan rate is 100 mV s^{-1}.

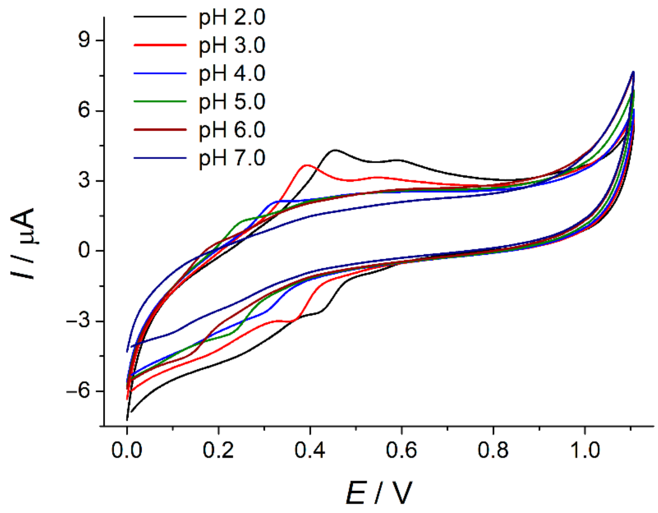

Figure S2. Cyclic voltammograms of poly(*p*-ABA)/MWCNTs/GCE in Britton-Robinson buffer at various pH. Potential scan rate is 100 mV s^{-1}.

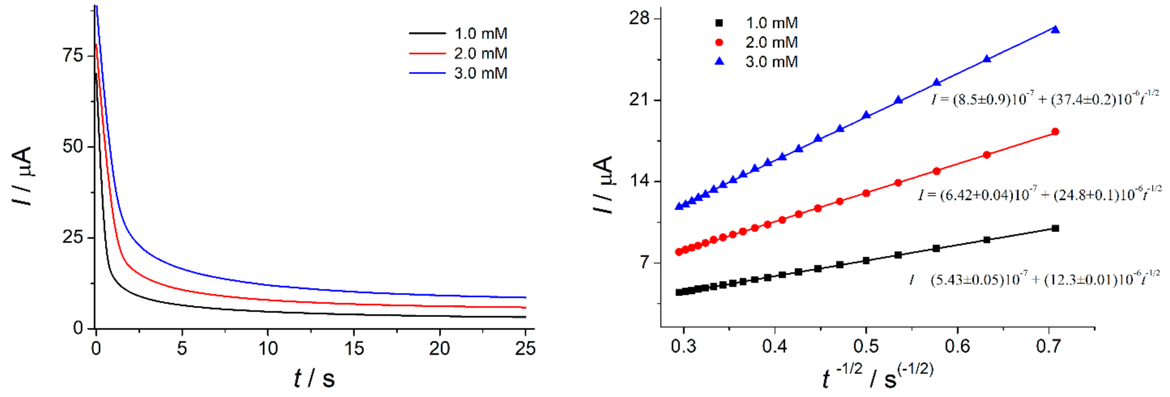

(**a**) (**b**)

Figure S3. (**a**) Chronoamperometric curves of 1.0-3.0 mM ferrocyanide ions in 0.1 M KCl at the bare GCE at 0.60 V; (**b**) Plot of *I* vs. $t^{-1/2}$ based on the chronoamperometric data.

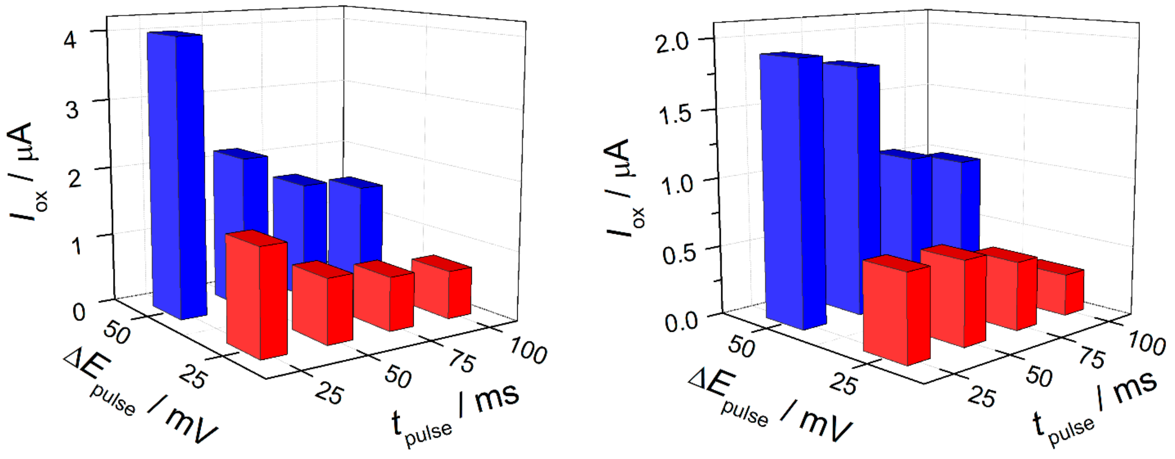

(**a**) (**b**)

Figure S4. Effect of pulse parameters on the oxidation currents of 10 μM mixture of aromatic aldehydes at the poly(*p*-ABA)/MWCNTs/GCE in Britton-Robinson buffer pH 2.0: (**a**) syringaldehyde; (**b**) vanillin.

Table S1. Figures of merit of electrochemical sensors for the simultaneous and individual determination of aromatic aldehydes.

Sensor	Method	Analyte	Detection limit (μM)	Linear dynamic range (μM)	Ref
Cetylpyridinium bromide/carbon nanofibers/GCE	DPV [1]	Syringaldehyde	0.19	0.75–10; 10–750	[63]
Poly(*p*-ABA)/MWCNTs/GCE	DPV		0.018	0.075–7.5; 7.5–100	This work
MWCNTs/GCE	DPV	Vanillin	3.44	4.15–294.12	[48]
Cathodically pretreated boron-doped diamond electrode	AdSWV [2]		0.38	3.3–330	[64]
Silver nanoplates/graphene/GCE	SWV [3]		0.332	2–100	[65]
Graphene/GCE	AdSWV		0.42	3.3–420	[66]
Graphene quantum dots@Nafion/Gold nanoparticle-modified SPCE [4]	DPV		0.32	0.66–33	[67]
Commercial MWCNTs/SPCE	DPV		1.03	2.5–750	[68]
Poly(methyl orange)/Graphene paste electrode	DPV		0.07	10–35	[69]
Poly(titan yellow) and octoxynol-9/CNT paste electrode	DPV		0.049	2.0–40	[70]
Poly(*p*-ABA)/MWCNTs/GCE	DPV		0.19	0.50–7.5; 7.5–100	This work

[1] Differential pulse voltammetry. [2] Adsorptive square-wave voltammetry. [3] square-wave voltammetry. [4] Screen-printed carbon electrode.

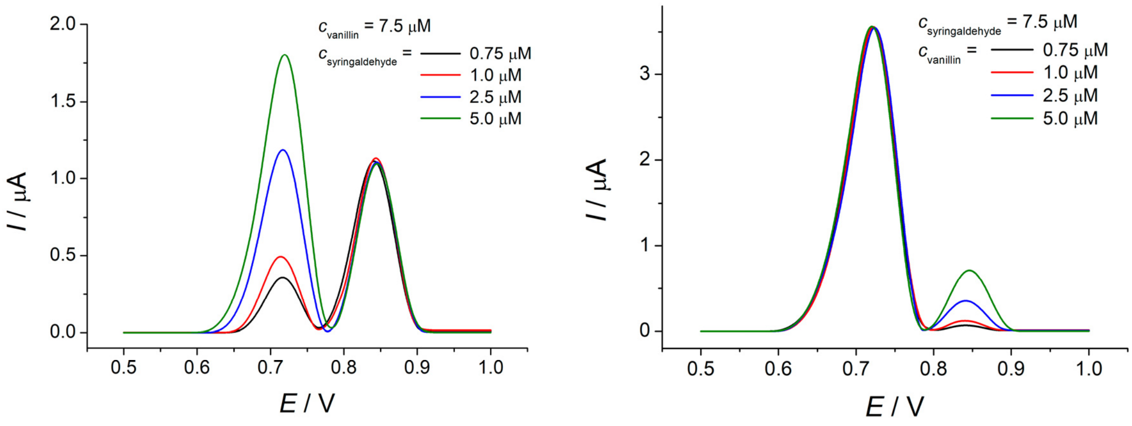

(**a**) (**b**)

Figure S5. Baseline-corrected differential pulse voltammograms of non-equimolar mixtures of aromatic aldehydes at the poly(*p*-ABA)-modified electrode in the Britton-Robinson buffer pH 2.0: (**a**) 0.75–5.0 μM of syringaldehyde in the presence of 7.5 μM vanillin; (**b**) 0.75–5.0 μM of vanillin in the presence of 7.5 μM syringaldehyde. Pulse amplitude is 50 mV, pulse time is 25 ms, potential scan rate is 10 mV s^{-1}.

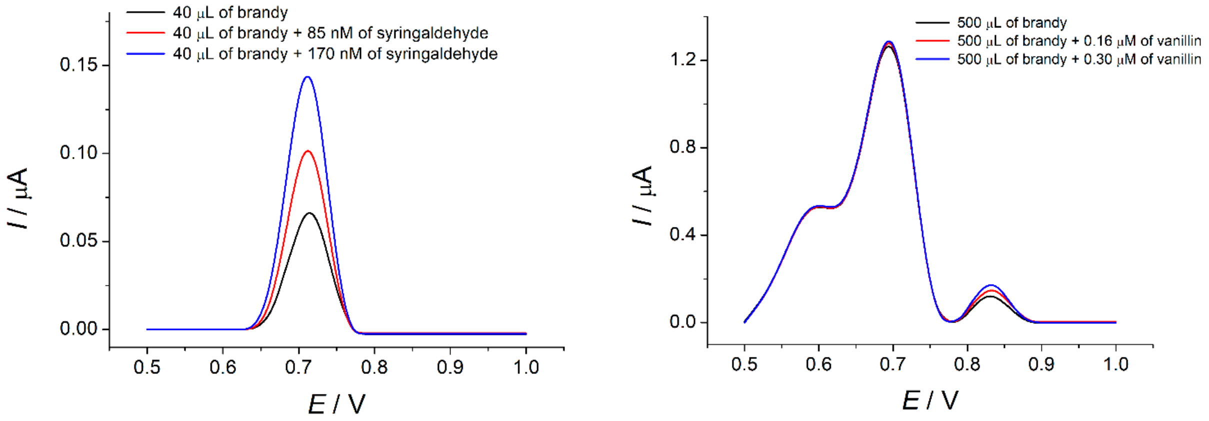

(**a**) (**b**)

Figure S6. Typical baseline-corrected differential pulse voltammograms of brandy at the poly(*p*-ABA)/MWCNTs/GCE in Britton-Robinson buffer pH 2.0: (**a**) 40 μL of brandy with various additions of syringaldehyde; (**b**) 500 μL of brandy with various additions of vanillin. Pulse amplitude is 50 mV, pulse time is 25 ms, potential scan rate is 10 mV s^{-1}.
